# Similar Trajectories in Current Alcohol Consumption and Tick-Borne Diseases: Only Parallel Changes in Time or Links Between?

**DOI:** 10.3389/fcimb.2021.790938

**Published:** 2021-12-16

**Authors:** Martyna Frątczak, Branislav Petko, Joanna H. Sliwowska, Jan Szeptycki, Piotr Tryjanowski

**Affiliations:** ^1^ Department of Zoology, Poznań University of Life Sciences, Poznań, Poland; ^2^ The University of Veterinary Medicine and Pharmacy in Košice, Košice, Slovakia; ^3^ Department of Preclinical Sciences and Infectious Diseases, Poznań University of Life Sciences, Poznań, Poland

**Keywords:** ticks, tick-borne diseases, mosquitoes, alcohol consumption, outdoor activity

## Abstract

In a modern world, both tick-borne diseases and alcohol consumption are among major public health threats. In the present opinion article, we pose the question, whether these two health problems: alcohol consumption and tick-borne diseases prevalence can be related. We hypothesize that it is possible due to at least three factors: outdoor places chosen for alcohol consumption, behavioral changes induced by alcohol, and possible stronger attraction of human hosts after alcohol consumption to ticks. Many important clues are coming from social studies about people’s preference of places to consume alcohol and from studies regarding the attraction of people consuming alcohol to mosquitos. These data, however, cannot be directly transferred to the case of alcohol consumption and ticks. Therefore, we suggest that more detailed studies are needed to better understand the possible individual attractiveness of people to ticks and ways alcohol may influence it.

## Introduction

Both tick-borne diseases and alcohol consumption are among major public health threats in the 21st century (Mansfield et al., 2017; WHO, 2021), whose importance has increased over the last decades. However, it is still puzzling if these two health problems changed only parallelly over the time axis, or are there any links between alcohol consumption and human attraction to ticks, and tick-borne pathogens? Thus, the main aim of this opinion paper is to indicate step by step potential interactions and then to discuss the above questions.

Ticks are second to mosquitoes as vectors of pathogens to humans and primary vectors of pathogens to domestic animals (Mansfield et al., 2017). The most important, from the human health perspective, species of ticks are representatives of Ixodidae family. The most common Ixodidae ticks in Europe, *Ixodes ricinus* and *Dermacentor reticulatus*, are known to transmit *Borrelia burgdorferi* s. l. causing Lyme borreliosis, *Anaplasma phagocytophilum* causing human granulocytic anaplasmosis, *Francisella tularensis* causing tularaemia, *Rickettsia* bacteria, causing spotted fever rickettsiosis, *Babesia* bacteria, responsible for babesiosis, as well as tick-borne encephalitis virus, Louping ill virus and Tribec virus (Medlock et al., 2013).

Another representative of Ixodidae, an aggressive tick feeding on humans, *Ambyomma americanum*, very common in many regions of the North America, is a host of such important pathogens like *Ehrlichia chaffeensis* and *Ehrlichia ewingii*, causing human ehrlichiosis, as well as *Rickettsia* bacteria (Mixson et al., 2006).

Moreover, *I. ricinus* and *A. amercianum* ticks were recently linked to the development in human hosts a tick-borne allergic disease known as the alpha-Gal syndrome. In this syndrome, the immune response to antigens present in the tick saliva is leading to the anaphylaxis response to subsequent tick bites, red meat consumption and even intake of certain drugs (Commins and Platts-Mills, 2013; Commins, 2020; Fischer et al., 2020; Mitchell et al., 2020).

The distribution and activity of many tick species are changing, and the prevalence of human-tick contact, and tick-borne diseases is increasing, especially within urban areas (Medlock et al., 2013; Sormunen et al., 2020). The changes in distribution of species, partly linked to climate changes, were noted for e.g., *Ixodes ricinus* and *Dermacentor reticulatus* in Europe (Alkishe et al., 2017) and *Amblyomma americanum* in North America (Dahlgren et al., 2016; Raghavan et al., 2019).

Moreover, it is also highly possible, that changing climate will lead in the future to expansion of novel, more exotic, tick species and pathogens they are carrying to Europe and North America, even more heightening the risk of developing tick-borne diseases (Ogden et al., 2021). Such events are already observed. For example, an African species of tick, *Hyalomma rufipes*, vector of dangerous Crimean Congo hemorrhagic fever virus (CCHFV), was noted in recent years in Greece and Italy (Lindeborg et al., 2012; Toma et al., 2021).

According to WHO data (WHO, 2021), alcohol consumption in developed and developing countries is constantly increasing. Globally, alcohol consumption causes 3 million deaths each year, leads to the development of many diseases and disabilities of millions of people. It is of particular interest for the current paper to explore if there are potential links between these two health risk factors: alcohol consumption and ticks’ prevalence to human hosts?

Here, we hypothesize that these two health threats: alcohol and ticks may be linked due to the following factors: [1] outdoor places commonly chosen for alcohol consumption, [2] behavioral changes induced by alcohol, that lead to unresponsible activities and [3] possible stronger attraction of people to ticks caused by alcohol consumption.

To our best knowledge, these issues have not been studied yet, which is a bit surprising considering the popularity of alcohol consumption worldwide and the importance of tick-borne diseases prevention. We provided in this opinion not only potential links between two suggested factors but also pointed out the need for well-developed research in this area, which could have very practical implications for tick-borne diseases prevention.

## Alcohol Is Often Consumed in Areas of Tick Occurrence

In recent decades, species of common hard ticks in Europe, such as *Ixodes ricinus*, have significantly expanded their area of occurrence (Daniel et al., 2003; Jore et al., 2011; Jaenson et al., 2012) and are more and more often found in urban green areas where human exposure to ticks is very high. Importantly, in urban tick populations, a high prevalence of ticks infected with pathogens such as *Borrelia burgdorferi*, Rickettsiales and tick-borne encephalitis virus has been detected (Biernat et al., 2014; Oechslin et al., 2017; Grochowska et al., 2020).

The dynamic shifts observed in the tick distribution and tick-borne diseases prevalence are associated with the factors such as changes in land use, forest management and urban planning, changes of climate and in the distribution of tick-host species, as well as changes in human activity and lifestyle, resulting in greater exposure of people to infected ticks (Medlock et al., 2013). The latter aspect may be directly related to alcohol consumption. Social studies show that alcohol is very often consumed in green areas, also within cities. Drinking alcohol in green spaces is notably common among young people, especially exposed to the negative effects of alcohol consumption (Trell et al., 2014).

Although these observations can be treated as incidental, it is worth noticing, that green areas as a place of alcohol consumption and socializing became even more common during the ongoing Covid-19 pandemic, when in many countries under lockdown bars, pubs and restaurants were shut down. Interestingly, because of this, local authorities in some countries, like Canada, officially allowed drinking in city parks (City of North Vancouver, 2021; The Abbotsford News, 2021). It has been shown that people generally used green areas more intensively during pandemic than in previous years, even to the point of disruption of wildlife (Cukor et al., 2021).

Even if not taking Covid-19 pandemic to the picture, green areas are a very important space for human recreation (Stigsdotter and Grahn, 2011). Use of them may become even more popular due to the climatic changes, leading to prolongation of the warm weather season (Proebstl-Haider et al., 2021), causing people to have more contact with nature and dangers linked to it – like encountering ticks.

In most European countries, alcohol consumption is allowed from the age of 18, and in the United States from the age 21. The illegality of alcohol consumption by younger people leads many of them to experiment with alcohol outside the home. When they decide to drink alcohol, they usually choose secluded places and out of the reach of any form of external control (such as adults, police) (Percy et al., 2011; Townshend and Roberts, 2013). In one study, 29% of 15-year-olds admitted drinking outdoors, in city parks and similar locations (Hawkins, 2012). Several other studies also found (Valentine et al., 2010) that adolescents typically consume alcohol in so-called marginal public places, such as streets, bus stops, green spaces, and parks.

In the literature related to the threats caused by alcohol, different styles of its consumption are evident. One of them is the so-called binge drinking, i.e., episodic drinking in which huge amounts of alcohol are consumed in a short time, for example during the weekend. This pattern of alcohol consumption is common in groups of students who meet on weekends after a week of studying (Erickson, 2018). Such a drinking style can be especially dangerous when happening outside, due to causing irresponsible behaviors.

Choosing to drink alcohol outdoors may have different risks than drinking alcohol in pubs, clubs, or residential buildings. These risks include exposure to ticks and tick-borne diseases. Alcohol influences the locomotor behavior and decision-making of people (Lang et al., 2003; Lorant et al., 2013). Individuals after consumption of alcohol may walk uncarefully and in a changed trajectory, walking around a larger space than sober individuals. It is also not uncommon for people after excessive alcohol consumption to fall or choose random places outside to rest or sleep. In this way, the risk of encountering a tick may be higher for drunken people.

It is worth noting, that infections with tick-borne diseases are very often recorded in mushroom pickers, who admit going to the forest under the influence of alcohol (Iowa State University, 2021). Although they are generally a group exposed to the contact with ticks, information about this additional health risk could encourage them to use better tick protection. Among social groups, homeless people are especially prone to both alcohol consumption problems and tick-borne diseases (Bransfield, 2018).

## Possible Stronger Attraction to Ticks After Alcohol Consumption

The behavior of ticks when selecting an appropriate host can be divided into two phases: the host-finding behavior and the host-discrimination behavior. The most common ticks in Europe, family Ixodidae, look for suitable hosts in a very distinctive way, climbing over vegetation and waiting for it with outstretched legs. Several physical and chemical stimuli (non-specific for a host, like CO_2_, body heat, vibrations) inform the waiting tick about a potential host passing by (Lees, 1948; Camin and Drenner, 1978). If a potential host encounters a tick, the arachnid climbs onto it and then starts foraging or falls to the ground and starts the host-finding phase again. The choice or rejection of the host is based on host-specific physical and chemical stimuli, like individual body odor (Camin and Drenner, 1978; Steullet and Guerin, 1992). Different, and very often described as aggressive, strategy of acquiring the host can be found in *A. americanum* and some other ticks from the order *Amblyomma*. They use an active hunter strategy, in which they emerge from the habitat and run toward the potential host, that occurred nearby (Parola and Raoult, 2001).

Ixodidae ticks have highly sensitive chemical receptors, detecting, among others, CO_2_ and NH_3_, phenols and other substances that make up the host’s odor (Lees, 1948). The most important sensory organ of the tick is the Haller’s organ. It is located in the front pair of tick legs, playing a role analogous to insect antennas. The Haller’s organ contains receptors that respond to subtle changes in CO_2_ concentrations in the environment (Steullet and Guerin, 1992) and a variety of other compounds found in the host’s odour (Steullet and Guerin, 1994; Leonovich, 2004). Haller’s organ has been suggested to play a role in the perception of other stimuli as well, e.g., infrared light (Mitchell et al., 2017). Ticks can respond to the environment also due to numerous sensory bristles in the body and on the legs, which detect olfactory and thermal stimuli as well as the level of air humidity (Lees, 1969; Waladde, 1977). The sense organs include also the so-called *areae porosae*, a pair of depressions at the base of the head in female Ixodidae ticks (Woolley, 1972; Hess and Vlimant, 1986). It is believed that hunger and the detection of certain chemicals in the environment is the greatest motivating force for ticks to find a suitable host. Only unfed ticks have been shown to exhibit behavioral responses to odors, which confirms these stimuli are active during questing behaviors (Leonovich, 2004).

Human hosts may become more attractive to ticks after alcohol consumption through at least several mechanisms. Alcohol causes stronger sweating, dilatation of blood vessels, increases body temperature, emission of body heat and C0_2_ (Malpas et al., 1990; Desruelle et al, 1996; Wolf et al., 1999; Yoda et al., 2005). This way stimuli attracting ticks can be much stronger in the case of drunken individuals. It has been shown, that especially for ticks using hunting strategy of seeking a host, like *A. americanum*, CO_2_ is an extremely strong attractant (Carr et al., 2013). People after alcohol ingestion, emitting more CO_2_, could be particularly prone to attack of these active parasites.

## Alcohol as a Remedy for Ticks?

When discussing links between alcohol and ticks, it is worth to notice, that some people use alcohol as traditional repellent of ticks. For example, in a study concerning Polish and Czech university students’ attitude toward tick-borne diseases (Nejezchlebová et al., 2016) it was shown, that some of them use alcohol to avoid tick bites (although it was not specified, if they use it externally or internally). Other home remedies for tick’s protection included vitamins from group B and yeasts, that can also be found in beer. The real efficiency of such practices in tick borne diseases prevention is however doubtful.

Some tick “natural” repellent products are herbal infusions in alcohol, intended to apply externally (Lupi et al., 2013). They are usually not considered as very effective, due to the fact that product containing alcohol permeate deep into the skin, which for the external repellent means faster loss of protection (Bissinger and Roe, 2010). It is also quite popular to use alcohol to remove ticks from the skin, with the expectation that arachnid will drown in it. However, much more effective is to simply pull the tick out with tweezers, an eventually later dispose the tick by putting it into alcohol (CDC, 2021).

Similarly, being under the influence of alcohol does not protect from ticks. The amount of alcohol in blood consumed by the tick will be not sufficient to kill the insect. It may work for a small fruit fly *Drosophila melanogaster* to protect itself from blood-borne parasites by ingestion of alcohol (Milan et al., 2012) but not for a human body. If the concentration of alcohol in human blood would be high enough to kill the tick, it would probably also contribute to the death of the host itself.

## Discussion

**Figure 1:**
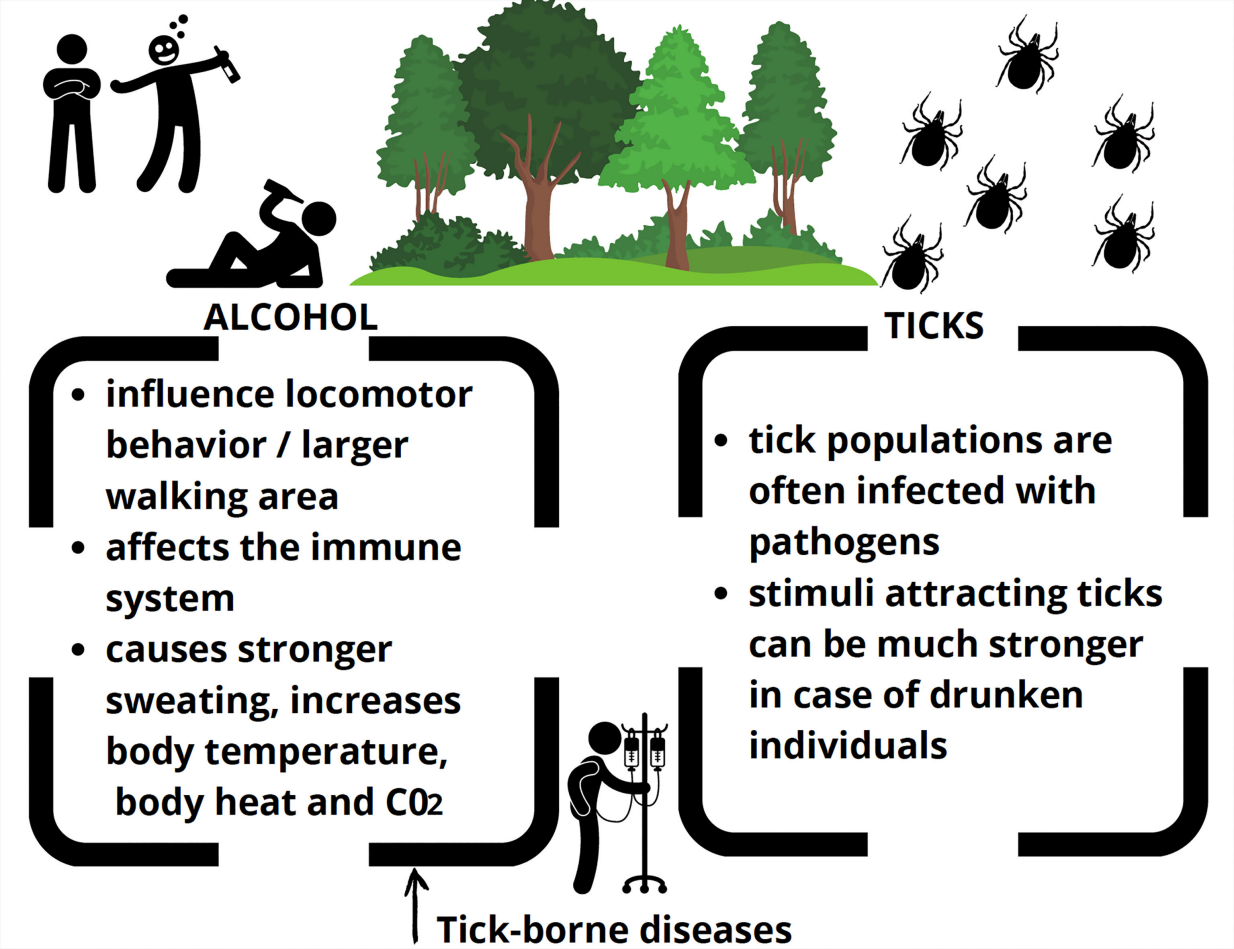
Graphical summary of the ideas described in the text.

The hypothesis that alcohol consumers may be attractive to ticks more than sober individuals has some basis in studies regarding other blood-sucking arthropods, reacting to similar stimuli as ticks (**Figure 1**).

It has been shown that people in short time after alcohol consumption attract more mosquitoes than those who do not (Shirai et al., 2002). The reason may lay in higher production of sweat, CO_2_ and greater emission of heat in the human body after alcohol consumption (Malpas et al., 1990; Desruelle et al., 1996; Wolf et al., 1999; Yoda et al., 2005). Both CO_2_ and heat generated by the human body are strong attractants for mosquitoes (Brown et al., 1951; Smart and Brown, 1956; Eiras and Jepson, 1994). Mosquitoes have also been shown to be attracted to increased sweat production (Khan et al., 1969; Maibach et al., 1996; Healy and Copland, 2000). Some researchers believe that increased exposure to mosquitoes after drinking alcohol is related to behavioral factors - people under the influence of alcohol may move more slowly and be easier to attack by mosquitoes (Shirai et al., 2002; Lefèvre et al., 2010). These effects of alcohol on humans could also influence the risk of contact with ticks.

Estimating the risk of human exposure to ectoparasites is usually based on the assumption that all people in the population are attractive to them to the same degree. However, observations and numerous studies show that it is not always the truth. Studies of *Anopheles* mosquito, a vector of malaria, bites prevalence show that humans differ greatly in their attractiveness to mosquitos (Brady et al., 1997; Kelly, 2001; Qiu et al., 2006). One study showed that only 20% of the population is responsible for more than 80% of all malaria infections in African children (Smith et al., 2005).

Each person has a characteristic body odour that is a result of the emission of more than several hundred volatile organic compounds present in the breath and emitted by the skin (products of glandular secretions interacting with skin bacteria) (Roberts et al., 2005; Lenochova and Havlicek, 2008). Additional factors such as diet, overall health, or even reproductive status and endocrine system functions may also influence an individual’s olfactory signal (Lindsay et al., 2000; Roberts et al., 2005; Havlicek and Lenochova, 2006). These factors are believed to be partly a cause of the observed varying degree of human attractiveness to malaria mosquitoes. For example, pregnant women are twice as attractive to *Anopheles* mosquitoes, and hence more likely to contract malaria, than non-pregnant women (Himeidan et al., 2004). Recent research on this topic has shown that beer consumption also affects people’s attractiveness to malaria mosquitoes (Healy and Copland, 2000). The body odour of people who consumed beer attracted mosquitoes much more than the smell of people who did not drink beer.

Anecdotal observations suggest that, as in the case of attractiveness to mosquitoes, people’s attractiveness to ticks is also subject to variability in the population. So far, however, there has been little research into the individual attractiveness of people to ticks. It has been shown that under controlled conditions, ticks of the *Amblyomma americanum* species are attracted more to air exhaled by women than by men (Josek et al., 2019). It is possible then, that potential metabolic differences between men and women, or sex hormones, play an important role in making an individual more attractive to ticks, but it needs more study in the future.

A review of the available literature indicates a strong need for studies focused on links between alcohol consumption and tick bites and tick-borne diseases prevalence in the human population. There are many possible ways in which these health problems can be related. Detailed experiments are needed to understand possible individual attractiveness to ticks and other vectors and ways the alcohol may influence it. An additional comparison worth a greater understanding is sex-related difference in the attractiveness of people to ticks and mosquitos.

Although alcohol is linked to the much more obvious dangers (like car accidents or developing liver problems), it could be beneficial for preventive medicine to find out, if it is also a risk factor of tick-borne and other vector-borne diseases. Awareness of such possibility could encourage people to better protection from ticks, when enjoying time in green areas.

## Data Availability Statement

The original contributions presented in the study are included in the article/supplementary material. Further inquiries can be directed to the corresponding author.

## Author Contributions

All authors reviewed the current knowledge, included their views of the discussed topic, edited, and read the manuscript. All authors contributed to the article and approved the submitted version.

## Funding

Research conducted by the statutory funding No. 506.511.05.00 of the Faculty of Veterinary Medicine and Animal Science Poznan University of Life Sciences, Poland; Department of Zoology (MF, JHS, JS, and PT). This work was supported by the Slovak Research and Development Agency under the Contract no. APVV-19-0440 (to BP).

## Conflict of Interest

The authors declare that the research was conducted in the absence of any commercial or financial relationships that could be construed as a potential conflict of interest.

## Publisher’s Note

All claims expressed in this article are solely those of the authors and do not necessarily represent those of their affiliated organizations, or those of the publisher, the editors and the reviewers. Any product that may be evaluated in this article, or claim that may be made by its manufacturer, is not guaranteed or endorsed by the publisher.
